# *Strongyloides stercoralis* infection in the UK: A systematic review and meta-analysis of published cases

**DOI:** 10.1016/j.clinme.2024.100227

**Published:** 2024-07-14

**Authors:** Ali M. Alam, Cansu Ozdemir, Nada Reza

**Affiliations:** a Newham Hospital, Barts Health NHS Trust, London, UK; b GKT School of Medical Education, King's College London, London, UK; c Antimicrobial Pharmacodynamics and Therapeutics Group, Institute of Systems, Molecular and Integrative Biology, William Henry Duncan Building, 6 West Derby Street, University of Liverpool, Liverpool L7 8TX, UK

**Keywords:** Strongyloides, Parasites, Helminths, United Kingdom, Systematic review

## Abstract

Strongyloidiasis is a helminth infection where symptoms vary, and asymptomatic presentation is common. Chronic strongyloidiasis can cause a high mortality ‘hyper-infection’ in immunocompromised states. Understanding at risk populations and symptomology can guide screening and early treatment to reduce hyper-infection risk.

A systematic review of studies describing patients in the UK with strongyloidiasis pooled a total of 1,308 patients. Weighted pooled prevalence (WPP) of asymptomatic cases was 27.7% (95% CI 17.1–39.5%, I^2^ = 92%, *p* < 0.01). At-risk populations included migrants, returning travellers and armed forces personnel. The most common symptoms reported were abdominal pain (WPP 32.1% (95% CI 20.5–44.8%), I^2^ = 93%, *p* < 0.01), rashes (WPP 38.4% (95% CI 13.1–67.7%), I^2^ = 99%, *p* < 0.01) and diarrhoea (WPP 12.6% (95% CI 6.7–19.9%), I^2^=70%, *p* = 0.03). Symptomatology varied with cohort characteristics.

Although asymptomatic presentation is common, patients may present with abdominal pain, diarrhoea or rashes. A low threshold for screening symptomatic individuals in at-risk groups is required.

## Introduction

*Strongyloides stercoralis* is a helminth endemic to tropical and subtropical regions which infects humans after contact with larvae-contaminated soil.^1^^,^^2^ After initial infection, *S. stercoralis* larvae generated in the intestinal tract can reinfect the host through a process of autoinfection, causing a lifelong chronic infection. Up to 70% of patients with chronic infection may be asymptomatic, with raised blood eosinophil count often being the only indicator of strongyloidiasis.^3^ In patients with clinical manifestations, symptoms include mild gastrointestinal and respiratory complaints, as well as a characteristic skin rash (larva currens).^4^ Though chronic infection can be asymptomatic or mild, it has the potential to lead to a hyper-infection state – a life-threatening syndrome which has a reported case fatality rate approaching 95%.^5^ Hyper-infection commonly results from impaired host immunity (which may be iatrogenic), wherein exaggerated parasite dissemination occurs and causes complications including meningitis, renal and respiratory failure and disseminated intravascular coagulation and shock.^3^^,^^5^ Chronic strongyloidiasis can be readily treated with anti-helminth agents.^6^ Therefore, screening of populations at risk of harbouring chronic strongyloidiasis is vital to enable prompt treatment and eradication, averting the risk of hyper-infection.

The worldwide prevalence of strongyloidiasis is estimated to be 8.1%, with regions of sub-Saharan Africa, South America and South-East Asia being recognised endemic areas with prevalence exceeding 20%.^7^ In the UK, the frequency of strongyloidiasis is unknown. Cases are mainly reported in migrants, and it is estimated that up to one in five arriving to the UK from endemic regions may be affected by helminth infections.^8^^,^^9^ Hyper-infection cases in the UK have been reported in the literature in a variety of clinical settings,^10^, ^11^, ^12^, ^13^, ^14^, ^15^ and cases may rise given greater global travel and migration, as well as an increasing number of immunocompromised individuals (eg due to increasing immunosuppressive therapies, organ transplants and long-term corticosteroid use).

It is therefore important to screen at-risk populations in the UK for chronic strongyloidiasis. There are currently no studies that have systematically reviewed the reports of strongyloidiasis infection within the UK, and therefore there is a paucity of data to identify the groups most at risk. We aimed to evaluate the demographics, clinical features and outcomes of reported strongyloidiasis in the UK through a meta-analysis of published studies. Our objective was to elucidate symptoms and characteristics that should increase suspicion and prompt screening of patients for strongyloidiasis in general medical settings (such as the acute medical unit and general practice).

## Methods

### Search strategy and selection criteria

Systematic review and meta-analysis was conducted according to the Preferred Reporting Items for Systematic Reviews and Meta-Analyses (PRISMA) guidelines.^16^ The review was registered in PROSPERO (CRD42023416600) prospectively.

Database searches of PubMed (MEDLINE), Scopus, Web of Science and Cochrane for English language peer-reviewed primary research articles published until 25 January 2023 were conducted. Search terms used a combination of the words ‘*Strongyloides*’ and ‘United Kingdom’. Full search terms can be found in Supplementary Table 1.

The Population, Intervention, Comparator, Outcome, Study Design (PICOS) criteria were used prior to selection (Table 1). All full-text studies reporting cases of *S. stercoralis* diagnosed in the UK were included, regardless of selective populations. Studies with case numbers below 10 and those not reporting demographic features of their cohorts were excluded to reduce sampling bias.

**Table 1:** PICOS inclusion criteria

Review question	What are the demographics, clinical features and outcomes of patients who are diagnosed with *Strongyloides stercoralis* in the UK?	
Population	All patients diagnosed with *S. stercoralis* in the UK.	
Intervention	Diagnosis of *S. stercoralis* through either: a) Stool microscopy or culture b) Serum serology	
Comparator	Social background (eg migrant or refugee populations; returning travellers); medical background (eg immunocompromised populations)	

Outcomes	**Primary**	**Secondary**
	Clinical features	Clinical features stratified by social and medical background
	Demographics of patients	Location of study
	Relevant investigation findings and diagnosis modality	
Setting	Studies taking place in any healthcare setting in the UK	
Study design	Randomised control trials, prospective cohort studies and retrospective cohort studies	

### Data extraction

Titles and abstracts from database searches were imported into EndNote X8 (Clarivate Analytics, Philadelphia, USA) and de-duplicated. Remaining studies were uploaded to Rayyan (Qatar Computing Research Institute, Doha, Qatar) for screening. Screening of titles and abstracts and subsequently full-text manuscripts was undertaken in parallel by two reviewers (AMA and CO) and conflicts were resolved through consensus. Data were then extracted by the two authors (AMA and CO).

For included studies, the following fields were gathered: publication year, study type, duration (months), location, cohort characteristics, number of patients with confirmed strongyloidiasis, and if available, sex ratios, average age, reported symptoms at diagnosis, diagnosis modality, average eosinophil count, treatment type, and outcomes (including presence of hyper-infection, death or successful treatment).

Included studies were split into subgroups according to the cohort characteristics:

• Mixed cohorts were studies which recruited from unspecified populations.
• Migrant cohorts were studies solely recruiting patients who have migrated to the UK.
• Returning traveller cohorts were studies recruiting patients who had returned to the UK from travel abroad.
• Armed forces personnel cohorts were studies recruiting patients who were returning armed forces personnel or returning prisoners of war.

### Quality assessment

Retrospective studies were classified according to the Newcastle Ottawa Scale^17^ and randomised trials were assessed according to the Cochrane Risk of Bias 2.0 tool.^18^

### Statistical analysis

Data analysis of descriptive statistics was performed using SPSS (Version 27, IBM, Armonk, USA). R statistics (Rstudio Version 4.0.1) was used to perform meta-analysis, and create figures, forest and funnel plots (ggplot2 and meta packages).

DerSimonian-Laird random-effects modelling was used to report weighted pooled prevalence (WPP) of clinical variables. A Freeman–Tukey double arcsine transformation was applied to our data. Subgroup analyses were performed. Forest plots were generated with natural scales, and heterogeneity was assessed using Cochran's Q test and I^2^, while Egger's test was used to test for publication bias.

Sensitivity analysis was performed to assess the effect of variables in our analysis. We separated studies which recruited from specific populations to adjust for the effect of mixing heterogeneous studies.

## Results

### Study characteristics and risk of bias assessment

**Fig. 1:**
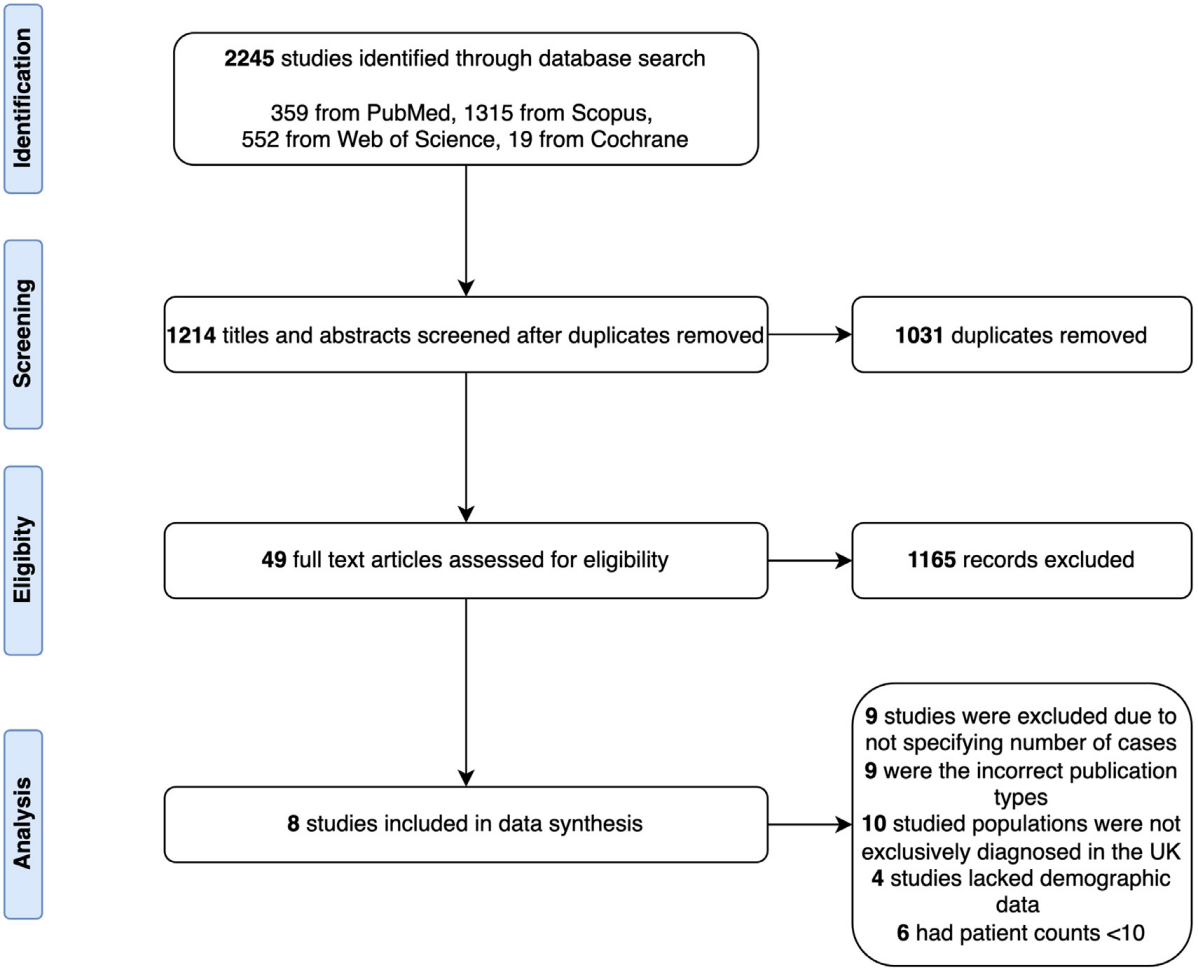
PRISMA flow diagram of study selection for inclusion in meta-analysis.

A total of eight eligible studies were included for meta-analyses^19^, ^20^, ^21^, ^22^, ^23^, ^24^, ^25^, ^26^ (Fig. 1). These included seven retrospective studies and one case–control study (Table 2). Publications were dated from 1978 to 2020, and six were based in London, while two were in Liverpool. The median (interquartile range (IQR)) length of study was 125 months (27.5–202.5).

**Table 2:** Included studies in meta-analyses. IQR = interquartile range, SD = standard deviation.

Study	Type of study	Location of study	Population type	Number of patients	Females (%)	Age – Mean (±SD) or Median (Range/IQR)	Diagnostic modality
Baker et al., 2020^19^	Case-control study	Borough of Tower Hamlets, London	Migrant cohort	115	53 (46.1%)	49 (IQR 38–61)	Serology
Barrett et al., 2017^20^	Retrospective study	Hospital for Tropical Diseases, London	Mixed cohort	121	47 (38.8%)	35 (Range 8–87)	Serology and stool microscopy or culture
Gill et al., 1979^21^	Retrospective study	Liverpool School of Tropical Medicine, Liverpool	Armed forces personnel cohort	88	0 (0.0%)	58 (Range 46–69)	Stool microscopy or culture
Gill et al., 2004^22^	Retrospective study	Liverpool School of Tropical Medicine, Liverpool	Armed forces personnel cohort	248	0 (0.0%)	65 (SD 7)	Serology and stool microscopy or culture
McGuire et al., 2019^23^	Retrospective study	Barts Health NHS Trust, London	Mixed cohort	100	29 (29.0%)	57 (SD 16)	Serology
Ming et al., 2019^24^	Retrospective study	Hospital for Tropical Diseases, London	Returning travellers cohort	413	158 (38.3%)	48 (IQR 36–61)	Serology and stool microscopy or culture
Sudarshi et al., 2003^25^	Retrospective study	Hospital for Tropical Diseases, London	Migrant cohort	192	60 (31.3%)	34 (Range 13–82)	Serology and stool microscopy or culture
Takaoka et al., 2016^26^	Retrospective study	Hospital for Tropical Diseases, London	Returning travellers cohort	31	8 (25.8%)	39 (Range 22–79)	Serology

Overall, 1,308 patients were included in our meta-analysis (Table 2). The mean (SD) age was 48.2 years (±11.4), and 355 (27.1%) of patients were female.

Of the 1,308 patients, 307 (23.5%) were recruited from migrant cohorts, 336 (25.7%) were armed forces personnel (including returning prisoners of war), 444 (33.9%) were returning travellers, with the remaining (221, 16.8%) from mixed cohorts.

Quality assessment revealed two studies had a high risk of bias, four had a moderate risk, and two had low risk. Full risk of bias assessment is detailed in Supplementary Table 2.

### Clinical features

**Fig. 2:**
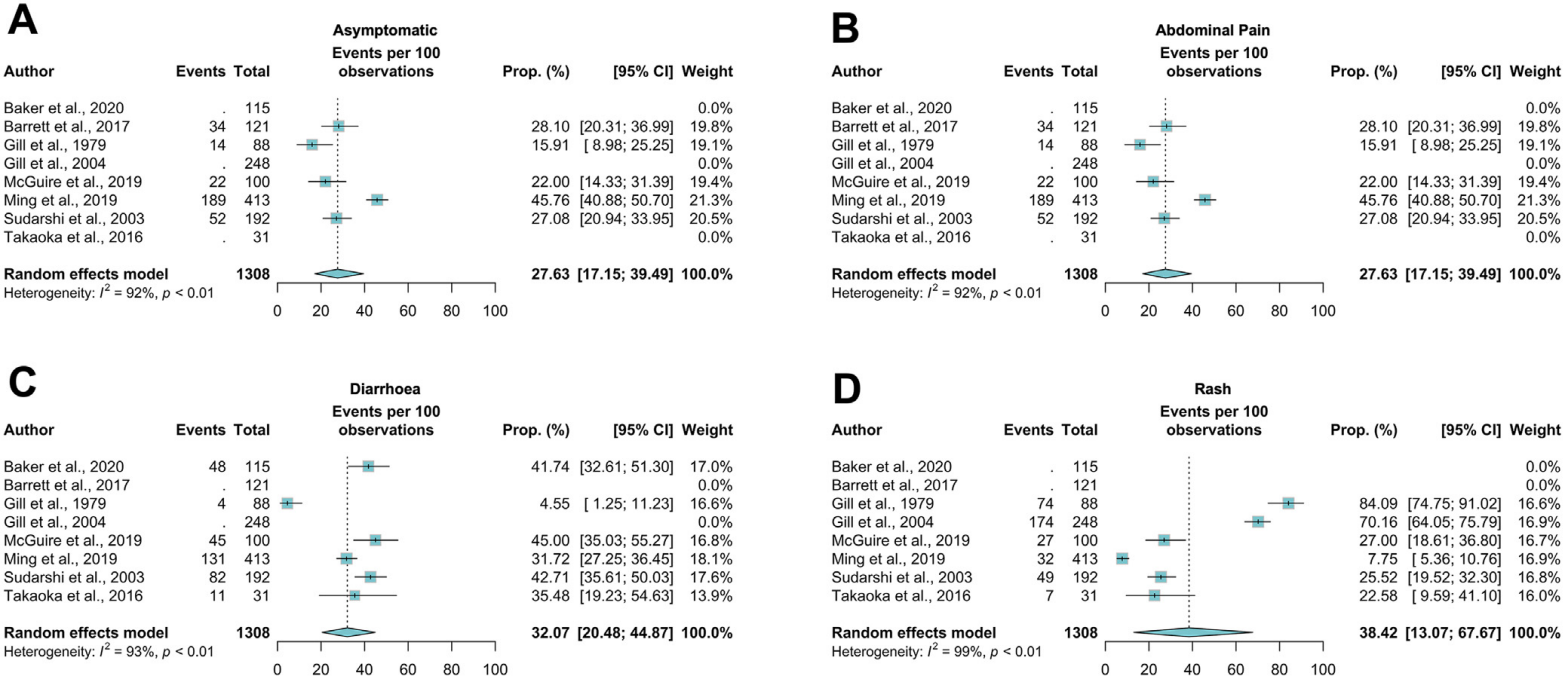
Forest plots showing prevalence of A – asymptomatic cases, B – abdominal pain, C – diarrhoea and D – rash. CI= Confidence intervals; Prop= Proportion.

We found a WPP of 27.7% (95% CI 17.1–39.5%, I^2^ = 92%, *p* < 0.01) for asymptomatic cases. The most common clinical features reported were abdominal pain (WPP 32.1% (95% CI 20.5–44.8%), I^2^ = 93%, *p* < 0. 01), rash (WPP 38.4% (95% CI 13.1–67.7%), I^2^ = 99%, *p* < 0.01) and diarrhoea (WPP 12.6% (95% CI 6.7–19.9%), I^2^ = 75%, *p* = 0.02) (Fig. 2). Funnel plots are available in Supplement 3.

### Sensitivity analysis

During sensitivity analysis (Table 3), the WPP of asymptomatic cases was higher when excluding armed forces (27.7% vs 30.7%), while the WPP was lower when excluding returning travellers (27.7% vs 23.7%).

**Table 3:** Sensitivity analysis of weighted pooled prevalence of symptoms in different cohorts with strongyloidiasis.

	Asymptomatic (% [95% CI])	Abdominal pain (% [95% CI])	Diarrhoea (% [95% CI])	Rash (% [95% CI])
*Whole cohort*	*27.7% [17.1 – 39.5%]* *I*Anon., *^2^ = 92%, P<0.0001*	*32.1% [20.5 – 44.8%]* *I*Anon., *^2^ = 93%, P<0.0001*	*12.6% [6.7 – 19.9%]* *I*Anon., *^2^ = 75%, P=0.0192*	*38.4% [13.1 – 67.7%]* *I*Anon., *^2^ = 99%, P<0.0001*
Excluding migrant cohort	27.7% [14.4 - 43.2%] I^2^= 94%, *P* < 0.0001	27.0% [11.2 - 46.5%] I^2^= 95%, *P* < 0.0001	10.6% [2.6 - 22.6%] I^2^= 86%, *P* = 0.0080	41.2% [9.7 - 77.3%] I^2^= 99%, *P* < 0.0001
Excluding armed forces personnel cohort	30.7% [19.6 - 43.0%] I^2^= 92%, *P* < 0.0001	39.1% [30.4 - 44.1%] I^2^= 65%, *P* = 0.0215	16.4% [10.1 - 23.9%] I^2^= *N*/A, *P* = *N*/A	19.6% [8.4 - 33.4%] I^2^= 93%, *P* < 0.0001
Excluding returning travellers cohort	23.7% [18.7 - 29.1%] I^2^= 47%, *P* = 0.1321	31.4% [13.5 - 52.7%] I^2^= 96%, *P* < 0.0001	12.7% [6.8 – 20.0%] I^2^= 75%, *P* = 0.0192	52.1% [23.6 - 79.9%] I^2^= 98%, *P* < 0.0001

CI= Confidence intervals.

The WPP of abdominal pain reduced more significantly when excluding migrant cohorts (32.1% vs 27.0%). The WPP of abdominal increased when excluding armed forces personnel (32.1% vs 39.1%).

The WPP of diarrhoea increased when discounting armed forces cohorts, although this only left one study reporting diarrhoea (12.6% vs 16.4%).

When excluding armed forces personnel, the WPP of rashes decreased significantly (38.4% vs 19.4%), whereas excluding studies recruiting returning travellers led to an increase in WPP of rash (38.4% vs 52.1%).

### Diagnosis, treatment, and outcomes

Serology was used for diagnosis in 677 (51.0%) patients, while stool microscopy or culture were used in 350 (26.8%). Where reported (*n* = 2), the mean eosinophil count was 1.45×10^9^/L (SD ±0.21×10^9^/L).

Where treatment was specified, 518 (49.1%) were treated with ivermectin and 174 (16.4%) with albendazole. Only two (0.2%) patients were reported to have hyper-infection syndrome.

## Discussion

This systematic review and meta-analysis explored the symptoms and characteristics of published strongyloidiasis diagnoses in the UK to help recognise which populations and presentations should prompt screening. Consistent with previous studies conducted in non-endemic areas, our findings highlight the diagnostic challenges associated with strongyloidiasis due to its tendency to either remain asymptomatic or present with non-specific symptoms.^10^^,^^27^^,^^28^ A quarter of patients with *S. stercoralis* included in our study were asymptomatic at diagnosis, while commonly reported symptoms included non-specific abdominal pain, diarrhoea and rash. Variation in presentation was observed between different cohorts.

### Case selection/sampling

The systematic review yielded at total of eight articles, conducted in either in London or Liverpool, likely due to the long-standing existence of tropical medicine institutions in both these locations. These centres provide services to a large geographical area of patients and have specialised parasitology laboratories. Therefore, although positive cases may be detected at these centres, they may have been referred from other UK regions. Nonetheless, there remains a need for further studies conducted in other populous urban areas with substantial foreign-born populations to facilitate better determination of appropriate indications for testing and treatment at a population level.^29^

### Asymptomatic infection

Over one-quarter (27.7%) of patients were asymptomatic at diagnosis. Asymptomatic infection occurs when mature helminths persist in the small intestine, maintaining a state of equilibrium with the host immune system.^30^ Within this balanced state, chronic strongyloidiasis results in granulocyte and eosinophil production.^30^^,^^31^

Approximately 70% of patients exhibit peripheral eosinophilia, which may serve as the sole basis for investigating strongyloidiasis.^32^ The proportion of asymptomatic cases we observed is comparatively lower than the reported figures in other non-endemic areas. A previous systematic review (which included five studies from Japan, Spain, Italy and the UK), found that 41.4% of diagnoses were asymptomatic.^32^ Among the studies included in these meta-analyses was a cohort study of 1,245 patients in Spain which reported that 82.1% of patients were asymptomatic at diagnosis.^33^ This difference may suggest that screening is less common in the UK. However, of the five included studies in the systematic review, diagnosis was primarily through combined serological testing and eosinophilia. In comparison, several of our studies included stool microscopy or culture in diagnosis. Numerous diagnostic tests are available for the diagnosis of strongyloidiasis, including stool examination, serological testing and PCR. Serological testing may overestimate the prevalence of the infection since it can remain positive even after the infection has resolved or cross-react with other helminth infections.^34^ This can lead to false-positive results and dilute the association of symptoms with strongyloidiasis.^32^ The sensitivity of microscopic diagnosis is improved by increased larval quantity, therefore prevalent use of microscopic diagnosis in our meta-analysis may skew our findings towards those with higher larvae burdens, which may in turn bias towards increased infection severity and symptomatic presentation. Additionally, our study included cases with co-infections, which introduces the possibility that positive symptoms were suggestive of other helminth infections.

There was heterogeneity when comparing asymptomatic infection between cohorts. Excluding returning travellers led to a decrease in the WPP of asymptomatic diagnoses, suggesting that this groups is being screened in some capacity.^35^ The presence of infective symptoms may depend on a high burden of larvae resulting from a significant period of autoinfection.^22^^,^^36^ This could explain the higher asymptomatic rates observed in returning travellers, who may have a limited opportunity for a high burden to occur due to relatively recent exposure to *S. stercoralis*.

In comparison, excluding armed forces personnel raised the WPP of asymptomatic diagnosis. This suggests this cohort is not screened as much as the others included in our study. Previous studies have shown that prisoners of war, for example, experience more severe helminth infection due to factors associated with their length in captivity, living condition and nutrition at the time of infection.^37^ Similarly, armed forces personnel have unique occupational exposures in endemic regions and are highlighted as a high-risk group.^38^ Given this, any patient who has served abroad in the armed forces should be considered for screening of strongyloidiasis in the UK, especially in the presence of eosinophilia.

### Symptomology

Exploring symptomology of chronic strongyloidiasis is important given that 30% may not exhibit eosinophilia.^32^ The most common symptoms reported in our analysis were abdominal pain, diarrhoea and the presence of a rash, which aligns with findings in other meta-analyses^32^^,^^39^ Presence of these symptoms should prompt screening in at-risk populations. Symptoms in strongyloidiasis arise when the balance between the host immune system and larvae production is disrupted. Host immune responses can vary for a multitude of reasons (eg genetic, environmental, comorbidity-related, microbiome-related).^30^ Unsurprisingly, differences in symptomology were seen between distinct cohorts.

Overall, 32% of patients reported abdominal pain at diagnosis, while 13% had diarrhoea. In sensitivity analysis, these rates may have been higher among the migrant cohorts. Migrants have an increased probability of experiencing concurrent infections that can lead to abdominal symptoms, such as giardiasis or amoebiasis. *S. stercoralis* detection in these cohorts may be incidental in association with symptomatic co-infections. Given the correlation between the burden of larvae migration through the gastrointestinal tract and amplification of symptoms, migrants may be more symptomatic due to longer time periods in endemic areas, increasing the risk of repeated *Strongyloides* larval exposure.^39^ Interestingly, this finding was not observed in the army personnel, which on sensitivity analysis showed lower rates of abdominal symptoms, despite extended stays in endemic settings allowing for significant larvae burden. Such differences could be due to variations in the human gut microbiome between cohorts. The gut microbiome can influence immune responses to helminths and parasites, and microbiomes differences may contribute to variations in gastrointestinal presentations among populations.^40^

Finally, 38.4% of patients reported a rash at diagnosis. Presentation with rashes was highest in armed forces personnel compared to other cohorts. This rash may have been larva currens or ‘creeping eruption’, which is often considered pathognomonic of strongyloidiasis, occurring due to larval migration through the skin resulting in a local hypersensitivity response. Genetic, immunological and environmental factors can influence hypersensitivity responses to parasites, and these may differ among ethnic groups.^41^ These variations may account for our findings; however, alternative infectious aetiologies for rashes in returning military personnel, may also result in the incidental diagnosis of strongyloidiasis.^42^

### Importance of screening immunocompromised cohorts

Hyper-infection is a significant risk in chronic strongyloidiasis. In our analysis, only two patients had reported hyper-infection. However, there was a paucity of data on the clinical presentation of strongyloidiasis in immunocompromised individuals, and further research in this subgroup of patients at the highest risk of hyper-infection is required.

Notably, one of the excluded studies in our analyses screened for strongyloidiasis specifically in people living with HIV, despite disseminated strongyloidiasis not being considered prevalent in advanced HIV disease.^43^ In comparison those living with human T-cell lymphotropic virus type 1 (HTLV1) are at significant risk, and though the rates of HTLV infection in England and Wales are low, most diagnosed cases are among individuals born outside the UK, increasing their risk of *S. stercoralis* infection.^44^ In Ming *et al*’s study, seven patients with HTLV-1 were included and it was noted that this sub-population may not present with peripheral blood eosinophilia, highlighting the need to understand symptomology suggestive of strongyloidiasis in the absence of increased eosinophils.^24^ More studies should focus on sub-population, and screening for HTLV-1 and strongyloidiasis should be routine when either is diagnosed.^45^

No included study explored chronically immunosuppressed individuals – eg those treated for autoimmune or haematological conditions or on transplant immunosuppression.^45^, ^46^, ^47^, ^48^, ^49^ In organ transplantation, most severe disease occurs within the first few months, and recent calls have been made to include screening in transplantation guidelines.^50^^,^^51^ Lastly, the documented cases of hyper-infection syndrome resulting from brief but intensive administration of corticosteroids in the context of COVID highlight the growing importance of screening for strongyloidiasis considering the expanding utilisation of immunosuppressive medications.^52^, ^53^, ^54^

### Limitations and future directions

This review is subject to certain limitations. Firstly, a limited number of studies provided detailed information on symptomology and demographics, which restricts the comprehensiveness of our findings. The possibility of selective reporting could have influenced the accuracy and completeness of our findings – especially in the army personnel cohorts which were both conducted in Liverpool.

Additionally, due to the scarcity of reported cases in the literature, we incorporated studies that examined co-infections, and this inclusion contributed to the heterogeneity observed within our analysis as these studies utilised different diagnostic approaches. Moreover, it is difficult to conclusively attribute reported symptoms to strongyloidiasis due to possibility the crossover in presentation with other similarly acquired parasitic and other infections.

Most included studies were conducted in a small number of distinct centres and in very limited UK regions. Although London possesses a higher population of individuals with origins from regions with *Strongyloides* endemicity, other known at-risk groups for strongyloidiasis may not be as well represented in this region. Given our findings of varied typical presentation in different at risks groups, sampling from other UK regions is essential to better elucidate possible indications for *Strongyloides* testing in an unselected cohort. Additionally, epidemiology in other urban areas (eg Leicester, Luton, Manchester) with significant migrant populations from *Stronygloides*-endemic areas have not been studied.^29^

In confirmed asymptomatic infection, studies of the prevalence of other clinical signs (particularly respiratory symptoms which were scarcely reported in the included studies) and positive biomarkers of disease (including eosinophilia) from a general cohort of patients will be useful given the high frequency of asymptomatic individuals who may eventually be at risk of hyper-infection. Additionally, existing datasets of population comorbidity and immunosuppression (eg for COVID-19 shielding and vaccination) could be utilised to identify those at risk of *Strongyloides* hyper-infection, the prevalence of undetected strongyloidiasis within this cohort, typical symptomatology, and other positive indicators for infection.

To better stratify the risk and presentation of undetected strongyloidiasis and hyper-infection in the general, further UK-wide studies are imperative with a view to improved guidance on screening and treatment.

## Conclusion

Our findings demonstrated that in a UK cohort, over one in four patients diagnosed with *S. stercoralis* may be asymptomatic, and presence eosinophilia in at-risk populations should prompt screening. The most common symptoms at diagnosis included abdominal pain, diarrhoea and rash. However, specific cohorts may have present differently at diagnosis, with those having extended periods in endemic region being most likely to present with symptoms. In these at-risk groups (eg migrants and armed forces personnel), non-specific abdominal pain, diarrhoea or rash with no other cause identified should prompt screening for strongyloidiasis. Given the risk of hyper-infection, clinicians should have a low threshold to screen any patient with exposure to endemic regions, especially if they are or will be considered for immunosuppression. Further high-quality evidence on strongyloidiasis in immunosuppressed cohorts, and specifically in those with HTLV-1, is needed (Box 1).

Box 1 **Summary of key practice implications for UK clinicians**

Key practice implications:

• Asymptomatic strongyloidiasis is common and screening should be considered in all individuals with a history of travel to endemic areas prior to immunosuppressive treatment to reduce the risk of hyper-infection.
• Symptomatology can differ based on patient characteristics.
• *Strongyloides* testing should be considered in migrants from endemic areas presenting with chronic gastrointestinal symptoms.
• *Strongyloides* testing should be considered in armed forces personnel with prior travel to endemic areas.

Alt-text: Unlabelled box

## Author contributions

AMA, CO, and NR contributed toward conceptualisation. AMA and CO contributed toward data curation. AMA completed formal analysis and writing - orginal draft AMA, CO, and NR contributed toward writing - review and editing. All authors confirm responsibility for the decision to submit the paper for publication.

## Declaration of competing interest

All authors certify that they have no affiliations with or involvement in any organization or entity with any financial or non-financial interest in the subject matter or materials discussed in this manuscript.

## Appendix Supplementary materials

Image, application 1
