# Supplementary material for: Strongyloides stercoralis infection in the UK: A systematic review and meta-analysis of published cases
Source: Clin Med (Lond). 2024 Jul 14;24(4):100227. doi: 10.1016/j.clinme.2024.100227 (PMC11342261; doi:10.1016/j.clinme.2024.100227)

**Supplement 1: Search strategies**

**Pubmed (MEDLINE) search strategy**

| **Search** | **Query** | **Results** |
| --- | --- | --- |
| #3 | #1 AND #2 | 367 |
| #2 | **(("united kingdom"[MeSH Terms] OR ("united"[All Fields] AND "kingdom"[All Fields]) OR "united kingdom"[All Fields] OR "UK"[All Fields] OR ("england"[MeSH Terms] OR "england"[All Fields] OR "england s"[All Fields] OR "englands"[All Fields]) OR ("united kingdom"[MeSH Terms] OR ("united"[All Fields] AND "kingdom"[All Fields]) OR "united kingdom"[All Fields] OR ("great"[All Fields] AND "britain"[All Fields]) OR "great britain"[All Fields]))** | 2173712 |
| #1 | **(("strongyloid"[All Fields] OR "strongyloides"[MeSH Terms] OR "strongyloides"[All Fields] OR "strongyloids"[All Fields] OR ("strongyloidiasis"[MeSH Terms] OR "strongyloidiasis"[All Fields] OR "strongyloidiases"[All Fields]) OR ("strongyloides stercoralis"[MeSH Terms] OR ("strongyloides"[All Fields] AND "stercoralis"[All Fields]) OR "strongyloides stercoralis"[All Fields]))** | 6860 |

**Scopus search strategy**

| **Search** | **Query** | **Results** |
| --- | --- | --- |
| #4 | #3 AND ( LIMIT-TO ( PUBSTAGE , "final" ) ) AND ( LIMIT-TO ( AFFILCOUNTRY , "United Kingdom" ) ) AND ( LIMIT-TO ( DOCTYPE , "ar" ) ) AND ( LIMIT-TO ( LANGUAGE , "English" ) ) | 1314 |
| #3 | #1 AND #2 | 6836 |
| #2 | ( "united kingdom" OR uk OR england ) | 18920015 |
| #1 | ( strongyloides OR strongyloidiasis ) | 19353 |

**Supplement 2: Bias Assessment**

| **Paper** | **Was the exposed cohort representative of the population at risk?** | **Was the cohort selected in an acceptable way?** | **0=poor, 1=fair, 2=good** | **Was the exposure/diagnosis from a reliable record?** | **Was there demonstration that outcome of interest was not present at start of study?** | **0 = poor, 1=fair, 2=good** | **Were the cohorts selected comparable?** | **0 =poor, 1=good** | **Were outcomes assessed appropriately?** | **Was follow-up long enough for outcomes to occur?** | **Was the adequate amount of people followed up?** | **0,1 =poor, 2=fair, 3=good** | **Total**  **6-8 good,**  **4-5 moderate, 3 and below poor** |
| --- | --- | --- | --- | --- | --- | --- | --- | --- | --- | --- | --- | --- | --- |
| Arkell et al., 2019 | ✔ | ✔ | 2 | ✔ | ✘ | 1 | ✘ | 0 | ✘ | ✘ | ✘ | 0 | 3 |
| Bailey et al., 2006 | ✘ | ✘ | 0 | ✔ | ✔ | 2 | ✘ | 0 | ✘ | ✘ | ✘ | 0 | 2 |
| Baker et al., 2020 | ✔ | ✔ | 2 | ✔ | ✘ | 1 | ✔ | 1 | ✔ | ✔ | ✔ | 3 | 7 |
| Barrett et al., 2017 | ✔ | ✔ | 2 | ✔ | ✘ | 1 | ✔ | 1 | ✔ | ✘ | ✘ | 1 | 5 |
| Fellner et al., 1978 | ✔ | ✔ | 2 | ✔ | ✘ | 1 | ✔ | 1 | ✔ | ✘ | ✘ | 1 | 5 |
| Gill et al., 1979 | ✘ | ✘ | 0 | ✔ | ✘ | 1 | ✘ | 0 | ✔ | ✔ | ✘ | 2 | 3 |
| Gill et al., 2004 | ✘ | ✘ | 0 | ✔ | ✘ | 1 | ✔ | 1 | ✔ | ✘ | ✘ | 1 | 3 |
| Gowland et al., 2022 | ✘ | ✔ | 1 | ✔ | ✘ | 1 | ✔ | 1 | ✔ | ✔ | ✔ | 3 | 6 |
| Harries et al., 1986 | ✔ | ✔ | 2 | ✔ | ✘ | 1 | ✔ | 1 | ✔ | ✘ | ✘ | 1 | 5 |
| McGuire et al., 2019 | ✔ | ✔ | 2 | ✔ | ✘ | 1 | ✔ | 1 | ✔ | ✘ | ✘ | 1 | 5 |
| Ming et al., 2019 | ✔ | ✔ | 2 | ✔ | ✔ | 2 | ✔ | 1 | ✔ | ✔ | ✔ | 3 | 8 |
| Roberts et al., 2003 | ✔ | ✔ | 2 | ✔ | ✘ | 1 | ✔ | 1 | ✔ | ✘ | ✘ | 1 | 5 |
| Sarner et al., 2007 | ✘ | ✔ | 1 | ✔ | ✘ | 1 | ✘ | 0 | ✘ | ✘ | ✘ | 0 | 5 |
| Sivaram et al., 2012 | ✔ | ✔ | 2 | ✔ | ✘ | 1 | ✔ | 1 | ✔ | ✘ | ✘ | 1 | 5 |
| Smith et al., 2010 | ✘ | ✔ | 1 | ✔ | ✔ | 2 | ✘ | 0 | ✘ | ✘ | ✘ | 0 | 3 |
| Sudarshi et al., 2003 | ✔ | ✔ | 2 | ✔ | ✘ | 1 | ✔ | 1 | ✔ | ✔ | ✔ | 3 | 7 |
| Takaoka et al., 2016 | ✔ | ✔ | 2 | ✔ | ✘ | 1 | ✔ | 1 | ✔ | ✘ | ✘ | 1 | 5 |

**Supplement 3: Funnel plots**


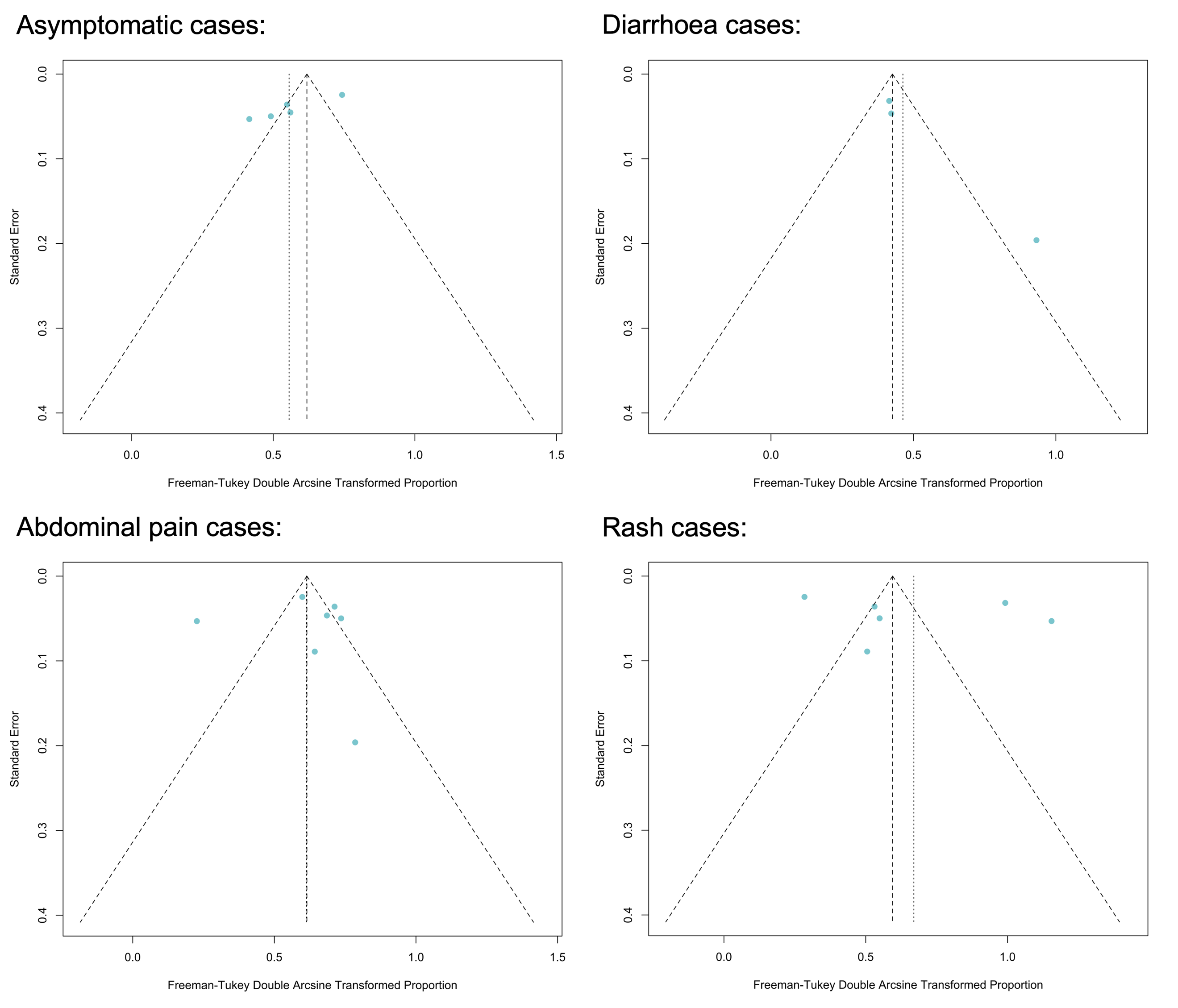

Supplement: Supplementary file 1 [file mmc1.docx]
